# Underpinning miRNA-miRNA co-functional interaction patterns in the metabolism of Oryza sativa by genome-scale network analysis

**DOI:** 10.1016/j.heliyon.2020.e05496

**Published:** 2020-11-12

**Authors:** Ayushman Kumar Banerjee, Chittabrata Mal

**Affiliations:** Amity Institute of Biotechnology, Amity University Kolkata, Major Arterial Road (South-East), AA II, Newtown, Kolkata, West Bengal, 700135, India

**Keywords:** Bioinformatics, Plant biology, Systems biology, Transcriptomics, Regulation, Rice, miRNA-miRNA co-functional network, Pathway, Module

## Abstract

MicroRNA (miRNA) is a class of non-coding small RNAs, which post-transcriptionally regulate a large number of genes and are now known to be important regulators in a wide variety of biological processes including metabolism. Thus, for better understanding these complex biological networks, and to derive their significance and inter-dependency, a systems biology approach enables us to explore and draw vital insights into these molecular network architectures. In this study, we aimed to understand the significance of synergistic miRNA-miRNA interactions in rice by constructing and analysing metabolic networks. The construction of the network involves target gene prediction of experimentally verified miRNAs of rice and then appending associated metabolic pathways to the network. A genome-scale miRNA-miRNA co-functional network (MFSN) is constructed based on co-regulatory interactions among the miRNAs and common target genes by applying transformational procedures. The analysis of the extracted MFSN modules identifies co-regulated target genes that are associated with corresponding interconnected metabolic pathways such as VALDEG-PWY (L-valine degradation I pathway was found to be targeted by multiple miRNA families, such as osa-miR812, osa-miR818, osa-miR821, and osa-miR5799 families while another pathway that was found to be associated with multiple miRNA families was PWY-6952 (glycerophosphodiester degradation pathway), PWY-6952 was found to be targeted by osa-miR812, osa-miR11344 and osa-miR5801 families. Such extensive study will help in systematically elucidating the regulatory networks in metabolism of rice, which in turn can be utilised to devise strategies for crop improvement and novel cultivar development.

## 1 Introduction

Rice (Oryza sativa L.) is among the most important species of grass and a staple food for almost half of the global population [1]. Our ever-increasing population and worsening climate have compounded over the years causing an overall decline in the amount of available cultivable land per person. This calls for the development of agricultural methods in addition to biotechnological intervention to be able to grow at least the essential crops, in indoor micro-environments using hydroponic technology [2]. At the same time, equal care must be taken to make sure the nutritional value of the crops is not adversely affected. To do this, a thorough understanding of physiological characteristics is required at a cellular level. This includes identification of target gene function, their regulation at the transcriptional and post-transcriptional level. Understanding and identifying metabolic pathways, their associated enzymatic activities, and the involvement of corresponding biomolecular interactions are a few key aspects in crop improvement and novel cultivar development which requires more research.

MicroRNAs (miRNAs) are a class of non-coding small RNAs, which are ~22 nucleotides in length [3] known to play important roles in regulating mRNA expression. The first microRNA (miRNA), lin-4, was discovered in 1993 by the Ambros and Ruvkun groups in Caenorhabditis elegans [4, 5] and has since revolutionized the field of molecular biology.

New miRNAs are continuously being discovered [6] and their roles in gene regulation are well recognized. MiRNAs play a critical role in normal animal development and are also involved in a variety of biological processes [7]. They have also been studied in several plants [8, 9]. Fundamental miRNA biology has been well-reviewed previously [10, 11, 12, 13]. Even though recent studies indicate that miRNAs play an important role in human metabolism [14], the same in plants is only moderately studied [15] and is mostly focused on stress responses [16]. Computational biology approaches in combination with bench research have been able to establish that miRNAs participate in metabolism functions in animals as well as in plants. Recent advancements in the field of molecular biology have shown that microRNAs (miRNAs) regulate vital metabolic processes in plants, typically at the post-transcriptional level [17, 18]. There are existing studies that elucidate conditional (stress) or pathway-specific regulatory involvement of miRNAs in plants but extensive metabolic network-based studies at genome-scale have not been done in rice – to the best of our knowledge. Systems biology approach and mathematical modelling of biological networks have shown much promise in understanding regulation of gene in complex biological processes when integrated with experimental techniques [19].

Owing to the recent increase in the availability of rice genome data derived from high-throughput experiments and algorithms, we have been able to explore the complex pairwise or synergistic relationships between miRNAs. In their study, Wu et al. found that 28 miRNAs can substantially inhibit p21Cip1/Waf1 expression [20]. This necessitates similar studies to be done on plants, especially in rice, due to its commercial importance. We focus on network analysis more compared to studying individual connections between miRNAs and their predicted targets, in this approach. Thus, studying pairwise or synergistic miRNA interaction is an important step towards determining miRNA functions at a genome-wide level.

Our understanding of synergistic interactions and its importance in domains ranging from biology to social sciences has increased over the years, and different methods have been developed to understand synergistic interactions that occur in biological networks [21, 22, 23, 24]. A randomization-based test devised by Balaji et al. [21] was used to identify pairs of transcription factors, in their study, that showed combinatorial regulation of target genes. Xu et al. [22] used network transformation to quantify synergistic associations between miRNA pairs with regards to their corresponding set of predicted targets, while Zhou et al. [23] established a miRNA-miRNA synergistic network by computing the pathway enrichment in the common set of genes for each miRNA pair. An et al. [24] used signal-to-noise ratio to identify regulating miRNAs for every gene and described a procedure to identify highly probable co-regulating miRNAs and their corresponding co-regulated sets of genes, which also involved several statistical tests. These studies, as mentioned, have clearly shown the significance of synergism in biological networks and have also indicated that integrating predicted targets with functional information could help in determining co-regulatory miRNA pairs and simultaneously reveal their underlying functional roles in the given networks.

**Figure 1:**
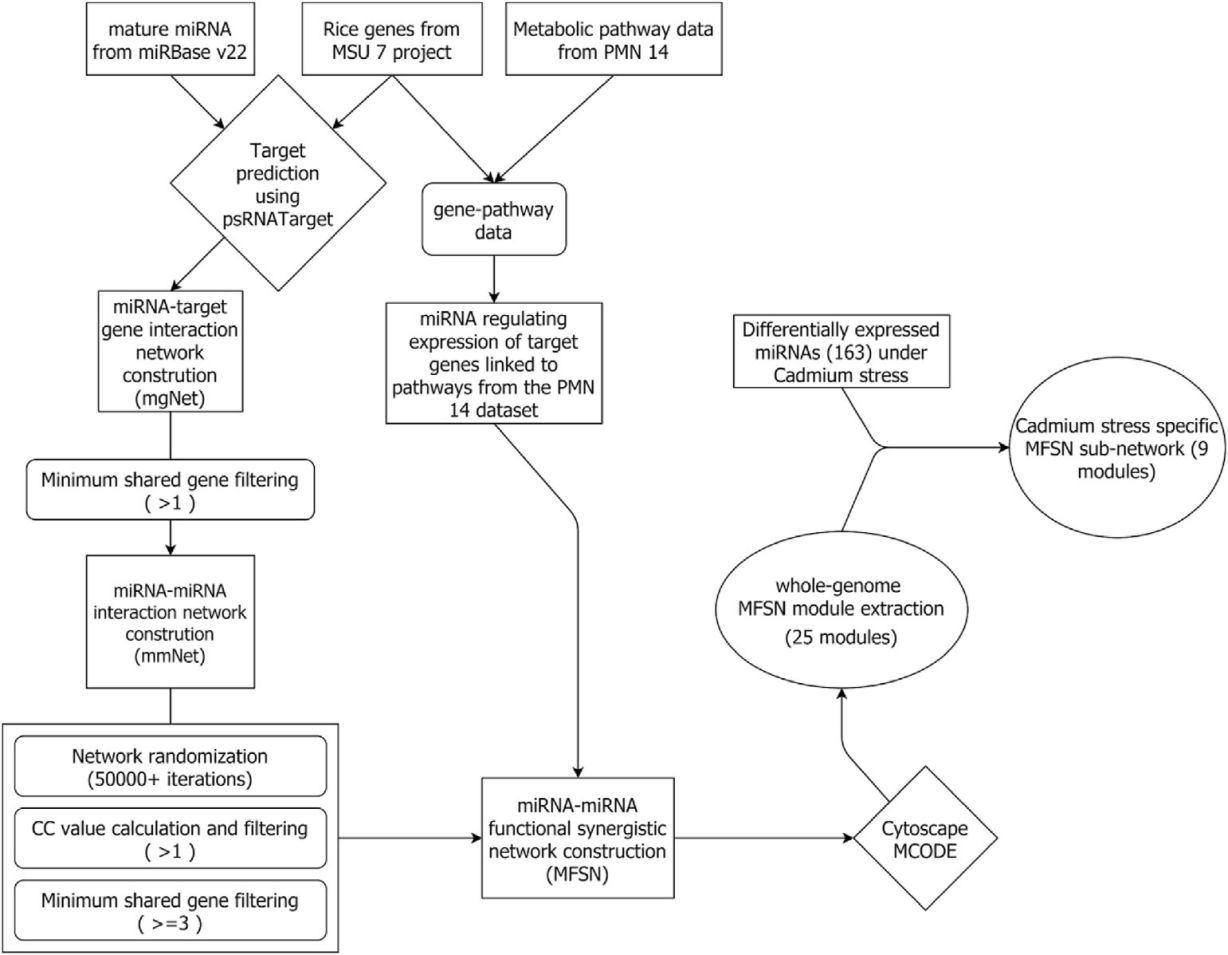
Overall workflow of creation of MFSN rice network and module analysis.

Based on these observations, in our present analysis, we have devised a computational method to identify pairs of miRNAs that show synergism derived via functional modules that they co-regulate by integrating predicted miRNA targets and their corresponding metabolic pathway information and transcriptional regulation by integrating corresponding data of transcription factors. Next, these miRNA pairs are further used for the construction of a miRNA-miRNA functional synergistic network (MFSN). Here, we defined a miRNA pair to be synergistic when it satisfies the following conditions: (i) co-regulated 3 or more target genes, (ii) had a co-regulatory coefficient of more than 1 (Figure 1 depicts the overall workflow of the analysis performed for this study).

Further, we examined the features of the MFSN organization derived at the end of our analysis using graph-theoretical methods and found it to be scale-free, modular, and a small-world network. This meant that the extracted network was distinct from the random networks and had the potential to be used to help understand miRNA synergism at a genome-wide level.

For validation of the predicted synergistic associations between the miRNA pairs, we used miRNA-mRNA expression profiles that were characterized by stress conditions. We also validated the synergistic features of the predicted miRNA pairs derived in the MFSN by studying literature on module pathways being interdependent or sometimes related, like both the pathways in module 22 are involved in ROS detoxification specific metabolism. We chose to base our work on predicted miRNA-target data, but we expect that more experimentally verified data will strengthen our approach in future. However, to minimize the counter effects of using predicted data, we made sure the prediction parameters were tuned so as to reduce false predictions as much as possible. Further, we integrated conditional expression profiles under cadmium stress with our results which also validated the findings of our study.

## 2 Materials and methods

### 2.1 Data retrieval

Metabolic data including pathways, reactions, enzymes, and genes were downloaded from the Plant Metabolic Network v14 [25] (https://www.plantcyc.org/) database. Sequences of mature miRNAs were obtained from the publicly available miRbase v22 (http://mirbase.org/) [26, 27]. Rice genome data was collected from Oryza sativa MSU Rice Genome Annotation Project v7 [28]. MSU LOC ID converted to RAP-DB ID for all the rice genes wherever applicable, and for the conversion of IDs, the latest mapping file was downloaded from the Rice Annotation Project Database (https://rapdb.dna.affrc.go.jp/download/archive/RAP-MSU_2020-06-03.txt.gz). Transcription factors for Oryza sativa were downloaded from the upgraded TF-centred database in PlantRegMap analysis platform (http://plantregmap.cbi.pku.edu.cn/) [29] and plnTFDB 3.0 (http://plntfdb.bio.uni-potsdam.de/) [30]. While the PRIN dataset was downloaded for the protein-protein interactions in rice (http://bis.zju.edu.cn/prin/) [31].

### 2.2 Software and tools

The plant-small RNA Target analysis server, psRNATarget [32] (http://plantgrn.noble.org/psRNATarget/) was used for the prediction of miRNA targets. Python 3 (https://www.python.org/) was used in this project for data processing. Cytoscape v3.8.0 [33] was used for visualizing all the networks. MCODE [34], a Cytoscape plugin, was used for module extraction. The rest of the network generation, extraction, and analysis were performed using Perl 5 (https://www.perl.org/).

### 2.3 Target analysis

The plant small RNA target analysis server (psRNATarget), an online tool, was used to predict miRNA targets. The 738 miRNA sequences downloaded from miRBase v22 were used as input for target prediction. The prediction was performed using the following settings taken from parameters as used by Nigam et al. for target prediction in rice [35] and by Lian et al. for target prediction in rice [36]: maximum expectation: 3.0; length for complementarity scoring (HSP): 20 bp; target accessibility: 25; flanking length around target site for target accessibility: 17 bp in upstream/13 bp in downstream and range of central mismatch leading to translational inhibition: 9–11 nt. Usually, target prediction algorithms have a tendency of generating false positives, and for the best possible representation, we have also cross-referenced our parameter settings with the constraints defined by Axtell et. al. for a reduction in false positives while using psRNATarget and found them to be in accordance [37]. All miRNAs, their converted IDs and corresponding predicted targets are provided in Supplementary File 1.

### 2.4 Network construction

#### 2.4.1 Construction of miRNA-target gene network (mgNet)

**Figure 2:**
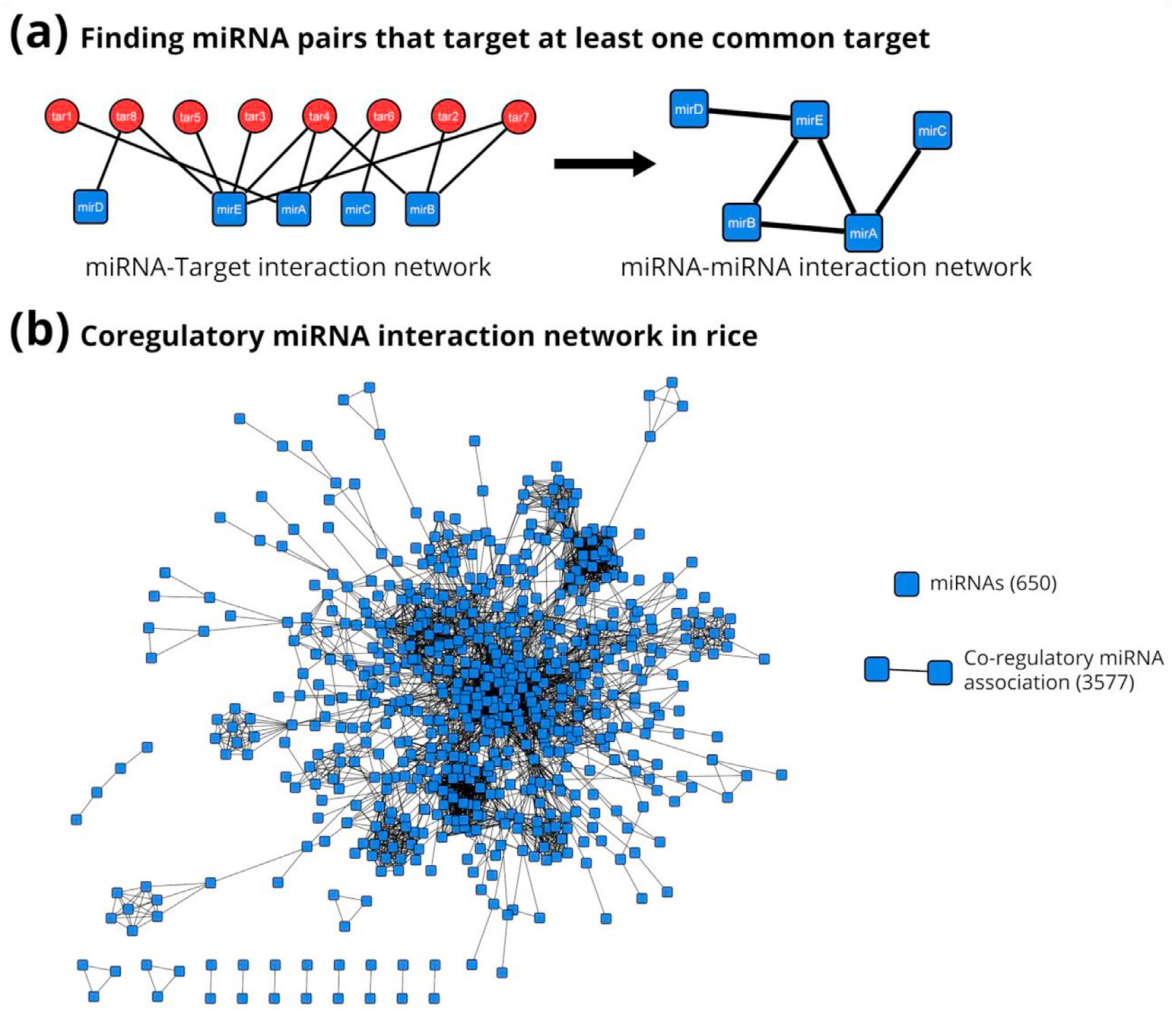
The procedure to determine a co-regulatory network starting from a miRNA-target interaction network. (a) Red circles represent target genes, blue squares represent miRNAs. Regulatory interactions are shown as black edges connecting target genes and the miRNAs in the network. The first step involves creating a network of miRNAs alone, where we have drawn lines between two miRNAs (black lines) if they target a common gene. (b) shows the initial co-regulatory miRNA-miRNA pairwise network formed as a result of the workflow depicted in (a), here too, blue squares depict miRNAs.

We start with the construction of the miRNA-target gene network (mgNet), where the miRNAs and their corresponding target genes are considered to be the nodes and each miRNA-target interaction is denoted via an edge. No restrictions or filters were applied for the construction of this genome-scale network of miRNA and target gene for rice. Figure 2(a) shows the network construction procedure and Figure 2(b) shows the initial coregulatory miRNA interaction network).

#### 2.4.2 Construction of preliminary miRNA-miRNA co-regulatory interaction network (mmNet)

For the construction of mmNet, we followed the methods prescribed by Balaji et al. [21], and an edge was initially established between a pair of miRNAs if they shared at least one common target gene between them. The number of miRNAs was reduced to 650 unique miRNAs because the other 88 miRNAs had no common target genes with the other miRNAs, and 3577 pairwise edges were identified among the 650 unique miRNAs. The number of common target genes was assigned to be the edge-weight for each of the miRNA-miRNA pairwise interaction edges. Next, we calculated the co-regulatory coefficient (CC) value for each pair of connected miRNAs by computing the ratio of the number of co-targeted genes by a miRNA pair in the original network to the average number of co-targeted genes by each miRNA pair for 50000 randomly generated miRNA-miRNA networks while maintaining the degree distribution for the number of shared targets by each miRNA pair. Finally, the updated CC values were assigned to the edges as edge-weights for each miRNA pair.

#### 2.4.3 Construction of miRNA-miRNA functional synergistic network (MFSN)

The miRNA pairs in mmNet which had a CC value less than 1 (i.e. observed co-functionality was lesser than the average CC value for randomly generated networks) were removed and only the pairs with values of greater than one were retained for further analysis. Next, G_min_ ≥ 3 (minimum number of shared or common targeted genes per pair) filter was applied to the network to obtain the final mmNet for the genome-scale analysis of rice metabolism. The value for G_min_ was chosen to be greater than or equal to 3 because on examination it was found that 3 was the median number of co-targeted genes by miRNA pairs in the mmNet, meaning, that at least half of the miRNAs were targeted by at least 3 or more target genes. The final mmNet was a sparse network with 660 edges and 195 nodes (miRNAs) which was derived after applying the following filters: (i) CC > 1; (ii) G_min_ ≥ 3. This network transformation method has been taken from Balaji et. al [21]. where they have also used the same CC value filter of greater than 1. The only deviation from the original method was that we carried out 50000 iterations of network randomization instead of 10000, to increase the stringency. The co-targeted set of gene(s) (m1∩m2) were extracted for each miRNA pair (m1 & m2) identified from the previously obtained miRNA-target gene network (mgNet). Only those miRNA pairs were used further which shared at least 3 common genes (Gmin ≥ 3) [21], and also had CC value >1. The probability (P) for the given m1 ∩ m2 was calculated as per the given equation:

$$P=1-F\left(n|T,\;G,L\right)=1-\;\overset{n}{\underset{t=0}{\sum }}\frac{\left(\begin{array}{c} T\\ t \end{array}\right)\left(\begin{array}{c} S\;-\;T\\ L\;-\;t \end{array}\right)}{\left(\begin{array}{c} S\\ L \end{array}\right)}$$

where T is the total number of target genes, G is the number of genes that are associated with their respective metabolic pathway and targeted by miRNAs, L is the size of m1 ∩ m2 (i.e. the number of co-targeted target genes by the given miRNA pair) and n is the number of targeted genes in m1∩m2 which is also annotated for the metabolic pathways. For FDR correction a cut-off value <0.05 was used with the Benjamini and Hochberg method. If a pair of miRNAs were involved in at least one path, they were considered co-regulatory, co-functional or synergistic. All the miRNA pairs identified as synergistic in this section were then assembled to construct a miRNA-miRNA functional synergistic network (MFSN). In this network, a single node represented a miRNA, and two nodes were joined if the corresponding pair of miRNAs had a synergistic relationship, otherwise, no edge was considered to exist between them.

#### 2.4.4 Identification of significant modules from MFSN using MCODE

**Figure 3:**
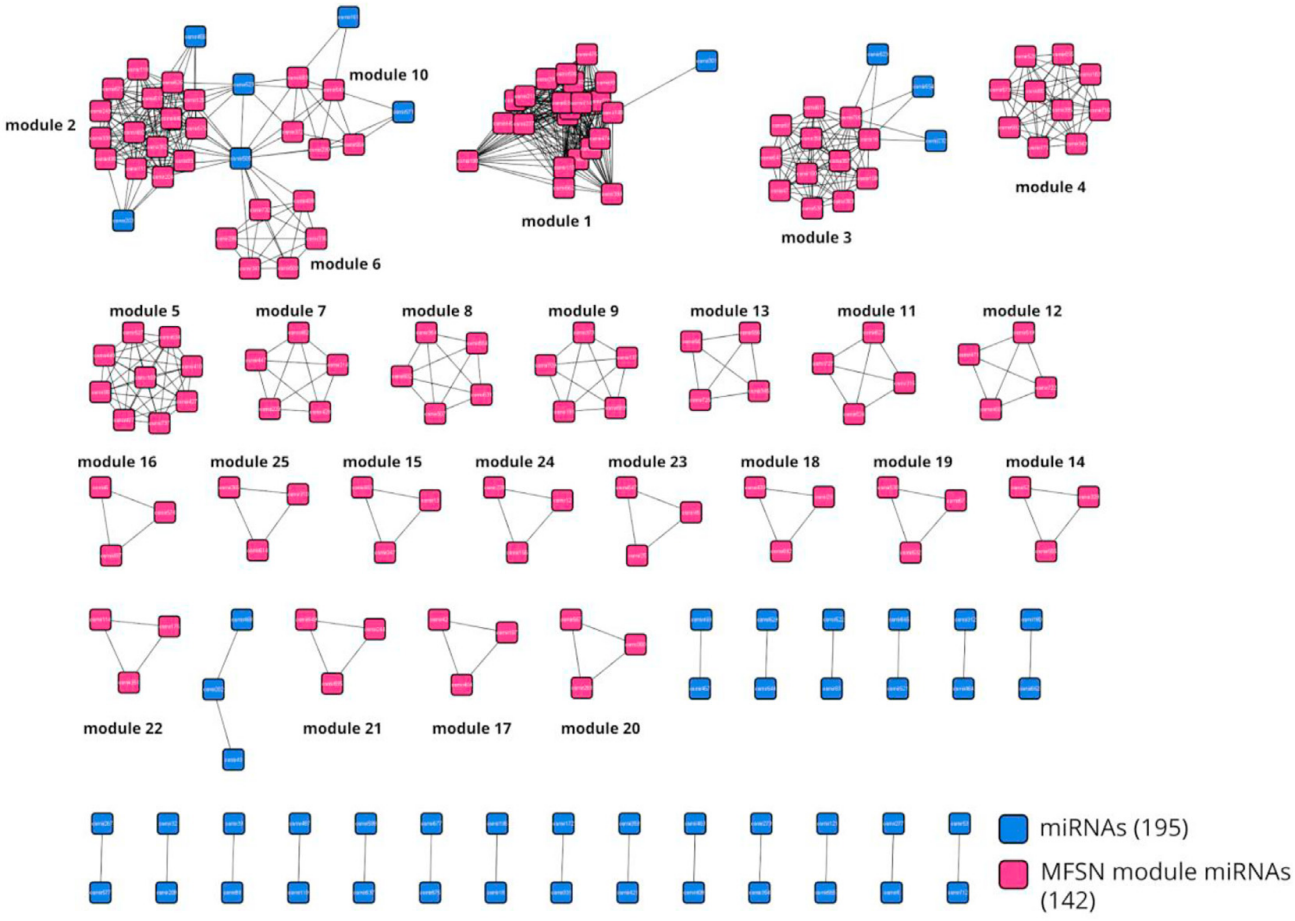
This shows the miRNA-miRNA functional synergistic network (MFSN); the miRNAs in this network are considered to be nodes (blue squares are miRNAs and dark pink squares are miRNAs that belong to modules extracted from MFSN via MCODE), and edges are drawn between a pair of miRNAs if there are at least 3 common target genes between them, and they have a CC value greater than one. The construction of MFSN is defined in section 2.4.3 and module extraction using MCODE is defined in 2.4.4. The clusters highlighted in dark pink are part of modules extracted using MCODE.

The Cytoscape plugin MCODE has been used to extract dense clusters from the MFSN network. The results obtained after running the MCODE algorithm on Cytoscape with the module specific miRNAs is provided in Supplementary File 2. MCODE defines a cluster as densely connected subgraphs in a given network based on topology [34]. Modules from the MFSN, in this study, have been defined as k-cliques, highly dense subgraphs with ‘k’ number of miRNAs where all miRNAs exhibit a synergistic association with the other miRNAs in the same subgraph. The MCODE plugin was run with the following default parameters: degree cut-off: 2; k-core value: 2; node score cut-off: 0.2; max depth: 100. Each extracted module (Figure 3 shows all the clusters obtained using MCODE) had a unique combination of miRNAs and none of the miRNA pairs were found to be present in more than one module. 142 miRNAs out of filtered 195 miRNAs were found to be in the twenty-five modules extracted using MCODE, i.e. 142 miRNAs were found to be significantly synergistic. And the remaining 53 miRNAs were not found to have any synergistic associations with the corresponding gene sets linked to metabolic pathways used in the present study.

## 3 Result and discussion

### 3.1 Network properties

#### 3.1.1 Network characteristics of the miRNA-target gene network (mgNet)

Considering the miRNAs and their corresponding target genes as nodes, and their interactions as edges, a post-transcriptional network (mgNet) of rice was constructed as described in section 2.4.1. This network had 5999 nodes (731 miRNAs and 5268 target genes) and 9780 edges. Each miRNA was found to target 13 target genes on an average (median = 9), while each gene was targeted by an average of ~2 (=1.856) miRNAs. The frequency distribution of miRNAs and their target genes showed that a large percentage of miRNAs (72%) target a comparatively lesser number of target genes (i.e. 1 to 15) in contrast to fewer miRNAs (28%) targeting 15 to 100+ target genes. Among all miRNAs in the network, the most connected was osmir347 (osa-miR2919), with 128 targeted genes while the most targeted gene was Os03g0743900 with 25 miRNAs.

#### 3.1.2 Network characteristics of the miRNA-miRNA co-targeting interaction network (mmNet)

For understanding the co-regulatory associations, a network transformation method was used allowing us to construct a co-regulation network (mmNet; Figure 2(b)) from the mgNet. As described in section 2.4.2, filtering out miRNAs that did not co-target even one gene with another miRNA were all removed, reducing the number of miRNAs in mmNet. With 650 nodes in this network, the expected number of possible co-regulatory associations between a pair of miRNAs was expected to be ^650^C_2_ (210,925) (^N^C_k_ = N!/[k!×(N–k)!). But on analyzing the network only 3577 (~2%) of the possible pair-wise co-regulatory associations were observed. This suggests that other combinations need to be explored to fully understand the co-regulatory association in rice miRNA network architecture. In this network, we also found that each node (miRNA) had an average of approximately 12 neighboring nodes (miRNA) and a global network clustering coefficient of 0.525. Accordingly, the rest of the focus was on studying the pair-wise co-regulatory associations.

### 3.2 General properties of the MFSN

As described in section 2.4.3 and 2.4.4, we constructed the MFSN based on miRNA pairs regulating a set of genes enriched in a particular metabolic pathway. The MFSN contained a total of 195 nodes (miRNAs) and out of a possible combination of 18,915 pairwise edges that can exist between 195 nodes only 660 edges (~3.5%) were found in the network. This makes the MFSN an objective representation of all synergistic associations between miRNAs. As for the network structure and organization, we observed that only a few miRNAs interact with a relatively large number of miRNA partners, whereas many miRNAs have lesser miRNA partners. On further examination it was found that the degree distribution of the MFSN is that of a power law distribution, showing that the MFSN is scale-free and extending the result of Reut Shalgi et al. [38], who identified miRNA co-operation among 64 miRNAs. Additionally, we also observed that the MFSN topology exhibited dense local neighborhoods even though not all the miRNAs were connected, forming sparse local clusters. Even then the MFSN had an average clustering coefficient of 0.849 which is much higher than for random networks (0.0563 ± 0.00471). We stipulated that this is because the immediate neighbors of the miRNA, its functional synergistic partners, tend to be synergistic. The dense neighborhood feature of small-world networks is particularly interesting because it can be exploited to predict synergism, as previously shown for protein-protein interactions [39].

#### 3.2.1 Characteristic features of the MFSN and features of the extracted modules

After analyzing the modular structure and community properties of the MFSN we defined modules in the MFSN to be k-cliques, i.e. highly dense subgraphs with k number of miRNAs where all miRNAs having a co-functional association with other miRNAs in the subgraph. Modules are identified using the MCODE plugin on Cytoscape as described in section 2.4.4. And as mentioned earlier, each module had a unique composition with a pair of miRNAs occurring only once in the network. The 142 unique miRNAs contained in all the 25 modules extracted via MCODE belonged to 32 different miRNA families. In total, out of 136 miRNA families in the initial data, only 32 families (23.5%) were present in modules extracted from the MFSN. We construed that miRNAs tend to perform certain regulatory functions in small clusters compared to acting individually or in bigger clusters. MiRNAs from the same family being involved in similar functions are obvious and, in this study, the majority of the modules (21 out of 25) had miRNAs from only one family and the remaining 4 modules had miRNAs from more than 1 miRNA family. We also found that out of the 32 families identified in the extracted modules, 61% of the 27 families containing 2 or more miRNAs were almost entirely part of at least one module. Therefore, we confirmed that miRNAs from the same family tend to be functional.

**Figure 4:**
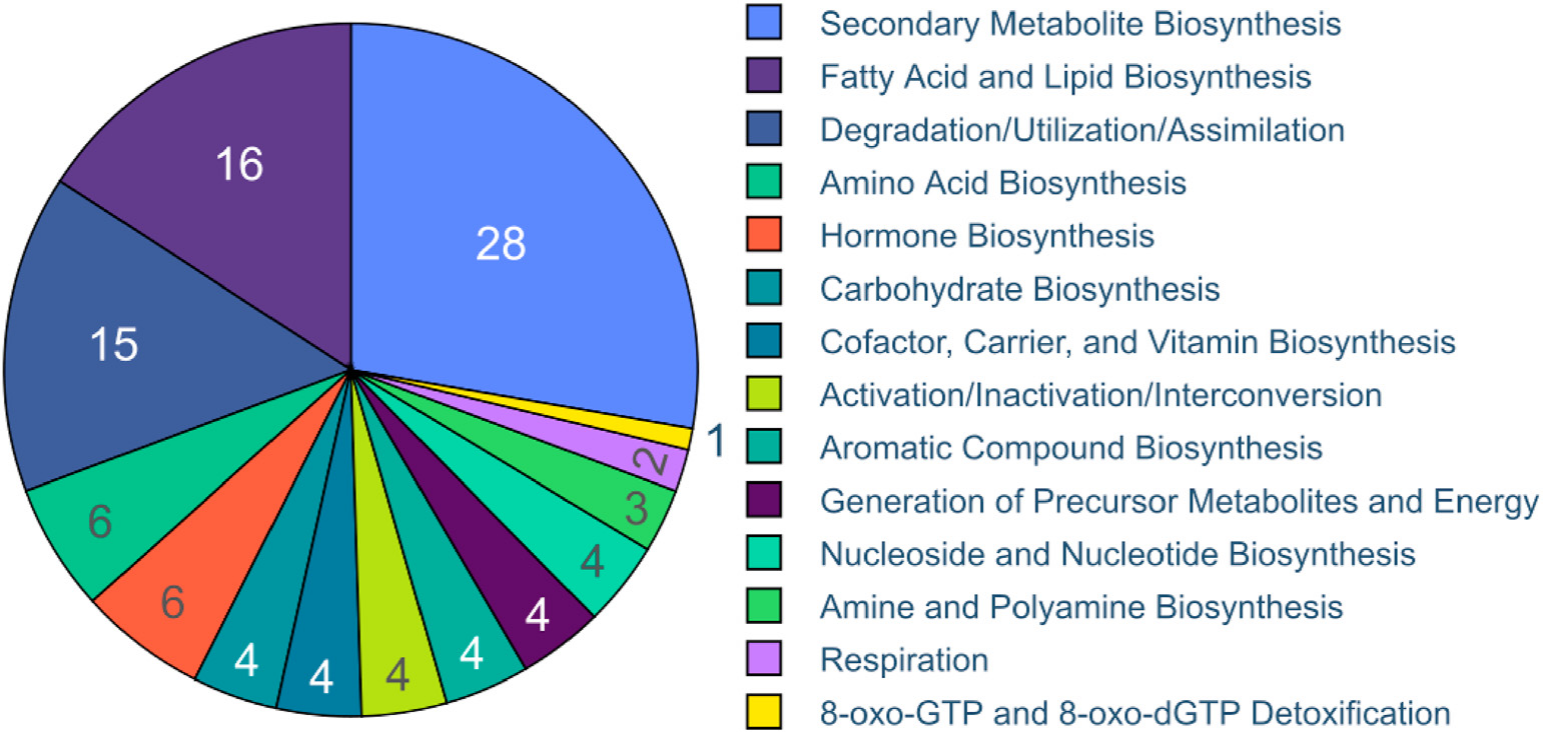
Pie-chart depicting distribution of different classes of metabolic pathways across all modules extracted from the MFSN. This diagram shows that the Secondary Metabolite Biosynthesis, Fatty Acid and Lipid Biosynthesis and Degradation/Utilization/Assimilation pathways are three major metabolic classes that are targeted by pairwise or synergistic miRNA pairs extracted from the MFSN module.

606 plant metabolic pathways were retrieved from PMN14 as described in section 2.1. This data was integrated with the MFSN network and after performing a pathway enrichment analysis, 91 (15%) out of 606 metabolic pathways were found to be in the 25 modules extracted from the MFSN using MCODE as described in section 2.4.4 (Figure 3 shows all the extracted modules using MCODE). These modules were composed of 142 miRNAs, 194 genes, and 91 pathways. Figure 4 shows the pathway class distribution in the MFSN. The largest module had 230 (22 unique miRNAs) pairwise associations whereas several other smaller modules were mostly composed of 3–9 (3–4 miRNAs) pairwise miRNA connections. On average, each module had 5 to 6 (5.68) miRNAs, ~9 (9.32) genes, and 4 pathways. On grouping all the miRNAs, we found them to be from 32 different families, and out of 25 modules, 4 were made up of miRNAs from more than one miRNA family. The significant miRNA pairs, their targeted metabolic pathways, and respective co-regulated genes are presented in Supplementary File 3. Certain overlaps in genes and pathways were identified between different modules. As for over-represented pathways, the L-valine degradation I pathway (VALDEG-PWY) was present in 3 different modules 2, 6, and 23. The overlapping modules in terms of gene and metabolic pathways have been provided in Supplementary File 4. Most modules were found to be associated with multiple pathways (e.g. module 10 in 17 pathways which were mostly secondary metabolite biosynthesis pathways like (PWY1F-FLAVSYN) flavonoid biosynthesis, (PWY-5466) matairesinol biosynthesis, (PWY-6275) β-caryophyllene biosynthesis, (PWY-6787) flavonoid biosynthesis, (PWY-7186) super pathway of scopolin and esculin biosynthesis, etc.), while other modules were involved in lesser pathways (e.g. module 13 was only involved in one pathway; (PWY-5129) sphingolipid biosynthesis).

#### 3.2.2 Role of transcription factors in MFSN

Next, we analyzed the transcription factors which are distributed with the targets of miRNA co-functional modules. Rice transcription factors (TFs) were downloaded from PlantRegMap (PlantTFDB 5) and plnTFDB 3.0 as described in section 2.1, and only the 1726 common transcription factors taken from both the datasets were used next. After integrating the TF data with the MFSN modules, there was no overlap found, meaning that the target genes associated with MFSN modules are not regulated by transcription factors. We found certain target genes from the mgNet that were present in the TF associated target list. Thus, we identified the miRNAs (296) of all the non-module TFs (338) and 86 miRNAs in the MFSN modules out of 142 miRNAs targeted some of the non-module TFs. 252 unique miRNA pairs made of these 86 miRNAs were found in the MFSN modules. On further analysis, we found that only 18 out of 25 modules were a part of this subnetwork of miRNAs that also targeted TFs that were not directly a part of the MFSN modules. For example, members of TF family MYB and MYB-related were co-targeted by modules 3, 15, and 20 by miRNA families 169, 159, 1858, and 2919. Wu et. al. had previously shown that osa-miRNA159 targeted mRNAs coding for MYB and other proteins that were involved in leaf senescence via phytohormone signaling pathways [40]. While members of TF family bZIP were co-targeted by the modules 11 and 12 by miRNA families 172 and 444. Zhou et. al. had experimentally found that miR172 and miR444 targeted genes encoding heat shock proteins that were associated with bZIP transcription factor while studying resistance to cold stress in Populus simonii × P. nigra [41].

However, in our study, these transcription factors have non-module target genes and do not show functional associations within the MFSN modules. Since the majority of MFSN modules are composed of miRNAs from a single-family, establishing a direct role of transcription factors in metabolic pathway regulation in rice did not seem feasible. Hence, we hypothesize that metabolic pathways in rice are perhaps regulated indirectly by TFs via the interaction between the synergistic miRNA pairs and the genes in the module and that TFs do not directly participate in metabolic pathway regulation. TF-network study by Gaudinier et. al. also showed that TF plays a central role in plant metabolism by regulating the expression of pathway-related enzymes [42].

#### 3.2.3 Role of identified miRNAs as principal module regulators in the rice metabolic pathway network

Often densely connected subgraphs in biological network analyses provide useful information, e.g. a dense protein interaction subnetwork may correspond to a protein complex [43, 44], and a dense co-expression network may represent a tight co-expression cluster [45]. Here, a similar approach was adopted to find miRNA hubs that could regulate and thus target a substantial number of target genes that were associated with several metabolic pathways. To achieve this, we used another Cytoscape plugin, called cytoHubba to identify hub miRNAs from the preliminary miRNA-Target gene network (mgNet). We used the MCC algorithm from cytoHubba, based on previous evidence of the MCC algorithm proving to be the most robust method in hub detection as shown by Chin et. al [46]. The only drawback is that MCC cannot find low-degree essential miRNAs. Given that there were 731 miRNAs in mgNet, we selected the MCC algorithm under Hubba nodes and chose the top 10% (73) miRNAs as hubs. This resulted in the identification of all the hub miRNAs and their list is provided in Supplementary File 5. On analyzing the extended subgraph containing all the identified hub miRNA, it was found that all the 73 miRNAs had 25+ score or outgoing connections. A sample t-test was performed (with α < 0.0001), which validated that the >25 connection to target genes was significantly greater than the rest of the miRNAs. Another statistic that was derived from this analysis was around 36% of the edges belonged to these top 10% of the miRNAs in the mgNet. Then, to make sure we were not automatically promoting the selection of miRNAs with maximum target genes, we also used EPC, a global-based algorithm on cytoHubba. Then we combined EPC predicted top 73 miRNAs and MCC predicted the top 73 miRNAs to identify 18 common miRNAs that were also present in the final MFSN network. A few of these miRNAs targeting 70+ target genes were: osa-miR11339-3p (osmir543), osa-miR1858b (osmir483), miR1858a (osmir13) and osa-miR11336-3p (osmir683). Hence, these hub miRNAs may have the potential to act as principal module regulators.

### 3.3 MFSN modules made up of multiple miRNA families

A few modules in the MFSN contained multiple miRNA families, as discussed in section 3.2.1. We picked these modules to study the targeted pathways under them to understand the interplay of two or more miRNA families behind the regulation of metabolic pathways due to pairwise interaction between miRNAs from more than one family. These 4 modules were mod:6, mod:10, mod:15, and mod:22 composed of miRNAs from 2, 3, 2, and 3 families respectively.

Module 10 was composed of 5 miRNAs from 3 miRNA families, 44 genes, and 17 pathways. 10 out of 17 were secondary metabolite biosynthesis pathways, for example, (PWY-5466) matairesinol biosynthesis, (PWY-6040) chlorogenic acid biosynthesis II, (PWY1F-FLAVSYN) flavonoid biosynthesis, (PWY-3101) flavonol biosynthesis, (PWY-6275) β-caryophyllene biosynthesis, etc. While pathways like (PWY-6030) serotonin and melatonin biosynthesis and (PWY-1822) indole-3-acetate activation I were hormone-related pathways. Similarly, for module 22, it was made up of 3 miRNAs, 3 genes, and 2 pathways. These pathways were (PWY-6502) 8-oxo-(d)GTP detoxification I and (PWY-102) gibberellin inactivation I (2β-hydroxylation). We found experimental evidence suggesting a relationship between these two pathways, because in a 2015 study done by Cheng et. al. suggested that the expression of genes in pathways associated with scavenging and detoxification of reactive oxygen species (ROS) were found to be substantially affected after gibberellin treatment [47]. While 8-oxo-(d)GTP detoxification I is a pathway involved in the elimination of potentially mutagenic nucleotides before they can be used for DNA replication or mRNA transcription [48] and is spontaneously induced when a hydroxyl radical attacks the C8 position of the purine base guanine [49]. Next, module 15 was composed of 2 miRNAs, 5 genes, and 5 pathways; (PWY-4861) UDP-α-D-galacturonate biosynthesis I, (HOMOSER-THRESYN-PWY) L-threonine biosynthesis, (PWY-5137) fatty acid β-oxidation III, (PWY-8011) L-serine biosynthesis II, (PWY-7861) N-hydroxy-L-pipecolate biosynthesis. Two of these are amino acid biosynthesis pathways for serine and threonine, and we found evidence in the literature that both these amino acids are directly involved in fatty acid oxidation pathways as suggested by the presence of PWY-5137 in module 15. Sim et. al. hypothesized that L-serine caused activation of Silent information regulator 1 (SIRT1) and as a result affected an increase in fatty acid oxidation [50]. Further, Ma et al. also observed a decrease in free fatty acid concentration followed by threonine supplementation even in a mouse model [51].

Based on these observations that pathways in a single module were always closely related or interdependent as suggested by experimental studies, we considered these experimental observations as evidence to suggest that the miRNAs for each module also acted in synergy or formed synergistic pairs due to their corresponding co-targeted pathways being related.

### 3.4 Distribution of differentially expressed miRNA under cadmium stress in MFSN modules

To further understand the implications of the results that we got, we used condition-based experimental data available for miRNA and mRNA expression profiles of rice. This allowed us to support our previous findings of miRNAs acting in synergy in the regulation of metabolic pathways, against publicly available experimental data.

**Figure 5:**
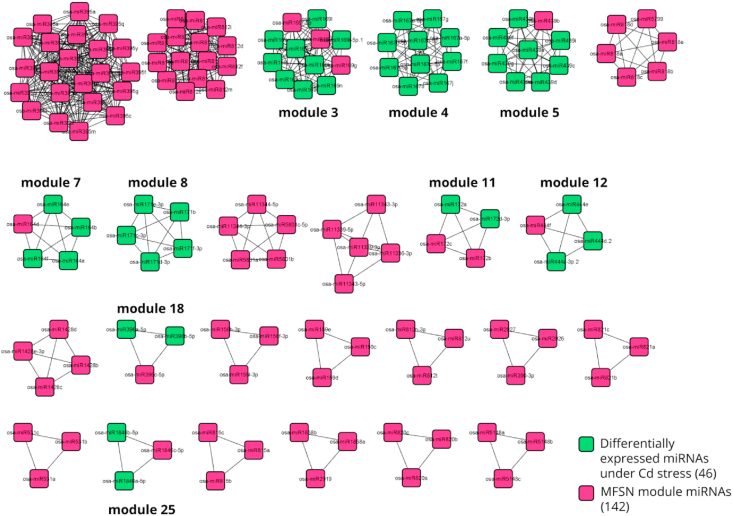
A list of miRNAs that were differentially expressed under Cd stress (green squares depict the miRNAs from MFSN modules that are differentially expressed under Cd stress while the dark pink squares show the miRNAs from MFSN modules that were not found to be differentially expressed under Cd stress or were not a part of the study) were taken from a study done by Tang et. al. [52], and then they were mapped onto the modules we extracted from the MFSN earlier. The overlapping miRNAs that were differentially expressed under Cd stress and also present in the MFSN modules are highlighted in green. They belong to 9 different modules out of 25; (3, 4, 5, 7, 8, 11, 12, 18 and 25) and are made up of 46 miRNAs from 9 different families.

In an integrated miRNA and mRNA expression profile analysis, Tang et. al [52]. identified 163 miRNAs that were differentially expressed under Cadmium (Cd) stress. They examined global genome expression changes caused due to Cd stress and its subsequent regulation done by miRNAs. Between these 163 differentially expressed miRNAs (DEMs) and the 142 miRNAs that were in the MFSN modules, we found 47 common miRNAs. Next, we calculated all possible pairs of these 47 miRNAs and matched them against all the synergistic pairs extracted in our MFSN modules. This gave us a subnetwork (Figure 5 highlights the miRNAs within the MFSN that are differentially expressed under Cd stress.), with only the synergistic miRNA pairs which were differentially expressed under Cd stress. We identified that this subnetwork consisted of 131 synergistic pairs made up of 45 unique miRNAs which were associated with 28 unique metabolic pathways.

These 131 pairs were derived from 9 different modules from the original MFSN which had 25 modules, namely: mod:3, mod:4, mod:5, mod:7, mod:8, mod:11, mod:12, mod:18, and mod:25. Eight pathways out of 28 identified in this subnetwork were secondary metabolite biosynthesis pathways like, (PWY-5048) rosmarinic acid biosynthesis I: osa-miR169 family, (PWY-7068) ursolate biosynthesis: osa-miR439 family, (PWY-7137) quercetin gentiotetraside biosynthesis: osa-miR164 family, (PWY-6351) D-myo-inositol (1,4,5)-trisphosphate biosynthesis: osa-miR171 family, etc. This was similar to the results obtained by Tang et. al. where they were able to identify enriched genes that encoded pathways involved in secondary metabolite synthases, signaling molecules, and ABC transporters along with ribosomal proteins and carotenoid biosynthesis. In addition to secondary metabolites pathways, we also found 8 fatty acid and lipid biosynthesis pathways in the subnetwork like: PWY0-1264, PWY-7388, PWY4FS-7, PWY-5269, PWY4FS-8, etc. Similar observations were made by Ammar et. al. where they had reported that the fatty acid biosynthesis pathways were disrupted due to the metallic stress caused due to cadmium treatment in tomato leaves [53]. On examining the DEMs obtained from the study done by Tang et. al., we found that the miRNAs like osa-miR444, osa-miR396, osa-miR167 were all downregulated both in shoot and root cells. And these miRNAs are present in the modules that contain the fatty acid biosynthesis pathways. This extends support to our analysis results obtained via MFSN module extraction and it shows that miRNA pairs expressed during stress conditions have the potential to regulate different biological processes.

The list of synergistic miRNA pairs expressed differentially under Cd stress, their corresponding modules, and associated pathways as predicted from the MFSN modules are presented in Supplementary File 6.

### 3.5 Future prospect and limitation

The analysis of co-functional modules, in this study allowed the identification of sets of miRNAs which exhibit the potential for post-transcriptionally alter known metabolic reactions via co-regulation. Additionally, following the approach of co-functional modular analysis, one can formulate hypotheses to examine possible correlations or interdependencies between several other metabolic processes and which can further be validated by performing relevant wet-lab experiments. It is essential to be mentioned that even though this co-functional network that was found in the identified in this study were supported by existing literature; one can show interest to utilize the latent relationships among other metabolic pathways, and reactions present within the same co-functional modules. One key drawback in these computational approach based research in biology is that most biological networks in the real words are sparse, and a few crucial disadvantages are (i) false or incorrect target prediction, and (ii) despite there being a substantial amount of genes which might be targeted by multiple miRNAs [54, 55] they might be non-functional in-vivo. Hence, experimental verifications for all derived hypotheses are required. Although a whole genome level miRNA-gene regulatory interaction network analysis may sound an extremely promising approach in understanding complex network architectures in nature, they are not easy to accomplish. However, recent studies have made the potential of miRNA-mediated gene regulation evident by demonstrating trait modification in plants via miRNA manipulation [56]. Therefore, the findings in this particular study might allow researchers in designing breeds of efficient and novel cultivars, with agronomically useful characteristics by manipulating pathways regulated by synergistic miRNAs.

## 4 Conclusion

In this study, we have re-constructed a genome-scale miRNA-miRNA synergistic network and have also integrated transcription factor regulatory data in the context of rice metabolic pathways. This has helped us identify important hubs/clusters of miRNAs from more than one family contributing towards regulation of metabolic pathways that fall under common functions. We also validated our findings using experimental data (differentially expressed miRNA and secondary metabolite pathway involvement under Cadmium stress). The methodology used in this study can be followed as a protocol to identify functional miRNA clusters responsible for regulation of rice metabolic pathways. In future, relevant experimental data will help in developing/improving our preliminary in-silico analysis to scan for metabolism-related miRNA clusters.

## Declarations

### Author contribution statement

Ayushman Kumar Banerjee: Performed the experiments; Analyzed and interpreted the data; Wrote the paper.

Chittabrata Mal: Conceived and designed the experiments; Analyzed and interpreted the data; Wrote the paper.

### Funding statement

This research did not receive any specific grant from funding agencies in the public, commercial, or not-for-profit sectors.

### Competing interest statement

The authors declare no conflict of interest.

### Additional information

No additional information is available for this paper.
