# Supplementary material for: Underpinning miRNA-miRNA co-functional interaction patterns in the metabolism of Oryza sativa by genome-scale network analysis
Source: Heliyon. 2020 Nov 12;6(11):e05496. doi: 10.1016/j.heliyon.2020.e05496 (PMC7672285; doi:10.1016/j.heliyon.2020.e05496)
Supplement: Supplementary Material [file mmc7.docx]

**Supplementary Materials**

Supplementary File 1: All miRNAs, their converted IDs and corresponding predicted targets.

Supplementary File 2: MCODE result with module specific miRNAs.

Supplementary File 3: The significant miRNA pairs, their targeted metabolic pathways, and respective co-regulated target genes.

Supplementary File 4: The overlapping genes and metabolic pathways in the MFSN modules.

Supplementary File 5: Hub miRNAs and their corresponding target genes.

Supplementary File 6: The list of synergistic miRNA pairs expressed differentially under Cd stress, their corresponding modules, and associated pathways.
